# *L1cam* curbs the differentiation of adult-born hippocampal neurons

**DOI:** 10.1016/j.scr.2020.101999

**Published:** 2020-09-17

**Authors:** Marta Grońska-Pęski, Melitta Schachner, Jean M. Hébert

**Affiliations:** a Departments of Neuroscience and Genetics, Albert Einstein College of Medicine, Bronx, NY 10461, USA; b Department of Cell Biology and Neuroscience, Rutgers University, Piscataway, NJ 08854, USA; c Center for Neuroscience, Shantou University Medical College, Shantou, Guangdong 515041, China

**Keywords:** L1cam, Neurogenesis, Dentate gyrus, Dendrite, Anxiety

## Abstract

L1 is an immunoglobulin domain (Ig)-containing protein essential for a wide range of neurodevelopmental processes highly conserved across species from worms to humans. L1 can act as a cell adhesion molecule by binding to other Ig-containing proteins or as a ligand for certain tyrosine kinase receptors such as FGFRs and TRKs, which are required not only during neurodevelopment but also in hippocampal neurogenesis. Yet, the role of L1 itself in adult hippocampal neurogenesis remains unaddressed. Here, we used several Cre-driver lines in mice to conditionally delete a floxed allele of *L1cam* at different points along the differentiation lineage of new neurons and in surrounding neurons in the adult dentate gyrus of the hippocampus. We found that *L1cam* deletion in stem/progenitor cells increased: 1) the differentiation of progenitors into new neurons, 2) the complexity of dendritic arbors in immature neurons, and 3) anxiety-related behavior. In addition, deletion of *L1cam* in neurons leads to an earlier age-related decline in hippocampal neurogenesis. These data suggest that L1 is not only important for normal nervous system development, but also for maintaining certain neural processes in adulthood.

## 1. Introduction

*L1cam* is an X-linked gene that encodes the neural cell adhesion molecule L1. *L1cam* is a member of the transmembrane immunoglobulin gene superfamily and one of four genes in the *L1cam* subfamily (*Chl1*, *Nrcam*, *Nfasc*, and *L1cam* itself). *L1cam* and its other subfamily members are widely expressed in the developing and adult nervous system (Allen Brain Atlas). L1 in particular is known to play essential roles during neurodevelopment (Kenwrick et al., 2000; Hortsch et al., 2014; Sytnyk et al., 2017).

The extracellular domain of L1 can interact with many other cell surface ligands and receptors and its intracellular domain can activate signaling cascades. In humans, engineered loss of *L1CAM* expression in ES-derived cultured neurons leads to deficits in axonal and dendritic arborization (Patzke et al., 2016). Over 350 inherited and spontaneous mutations in *L1CAM*, predicted to lead to loss of L1 function, have been identified in the human population (NIH, U.S. National Library of Medicine, Genetics Home Reference). These mutations often lead to “L1 syndrome”, almost exclusively in males due to the X-linked nature of *L1CAM*. Affected individuals exhibit a range of nervous system defects with the most common manifestations including hydrocephalus due to stenosis, spastic paraplegia type 1, abnormal gait, corpus callosum agenesis, aphasia, and intellectual disabilities (including autism and schizophrenia) (Wong et al., 1995; Christaller et al., 2017).

This spectrum of human nervous system defects overlaps with the spectrum observed in *L1cam* knockout mice and rats. *L1cam* knockout rodents exhibit hydrocephalus and enlarged ventricles, impaired hind limb motor control, reduced corpus callosum and corticospinal tracts, increased perinatal lethality, smaller body size, cerebellar defects, and other neurological deficits (Dahme et al., 1997; Cohen et al., 1997; Fransen et al., 1998; Emmert et al., 2019). The importance of L1 for nervous system development is conserved beyond vertebrates. In the worm *C. elegans*, for example, the L1 homolog SAX-7 is required for neuron positioning and neurite branching (Dong et al., 2013; Salzberg et al., 2013; Diaz-Balzac et al., 2015; Zou et al., 2016; Yang et al., 2019).

In worms and fruit flies, the growth and branching of axons and dendrites are in some cases mediated by an interaction between L1 and the homologs of Fibroblast Growth Factor Receptors (FGFRs) (Diaz-Balzac et al., 2015; Forni et al., 2004). An L1-FGFR interaction to promote neuron maturation extends to mammalian cells, as shown in several defined culture assays (Doherty and Walsh, 1996; Saffell et al., 1997) and in the proliferation and mobilization of glioblastoma cells (Anderson and Galileo, 2016). L1 can act as a ligand that activates not only FGFRs, but also neurotrophin receptors (Colombo et al., 2014). FGFRs and neurotrophin receptors play multiple roles throughout neurodevelopment (Guillemot and Zimmer, 2011; Hébert, 2011; Park and Poo, 2013). In addition, FGF and neurotrophin receptors are important in promoting multiple steps of hippocampal neurogenesis in the adult dentate gyrus (DG) where FGFR activity is necessary and sufficient to promote both neurogenesis and dendritogenesis and Tyrosine receptor kinase B (TRKB) activity promotes dendrite maturation (Kang and Hébert, 2015; de Vincenti et al., 2019).

However, the extent to which L1 plays a role coincident with that of FGFRs and TRKs in the adult DG remains unknown. Based on L1′s essential roles during neurodevelopment across species and in some cases its direct action through tyrosine kinase receptors, we hypothesized that L1 is required for one or more steps in the differentiation lineage of newborn neurons in the adult hippocampal DG. Using several Cre-driver mouse lines to conditionally delete *L1cam* in different cell types of the neurogenic lineage and surrounding neurons, we find defects in neuron production, dendritogenesis, and behavior, suggesting that L1 is critical not only during developmental neurogenesis, but also during adult hippocampal neurogenesis.

## 2. Results

### 2.1. L1cam deletion does not impact radial glia-like stem cell numbers

To begin examining potential roles for *L1cam* in adult DG neurogenesis, we crossed a previously validated conditional floxed *L1cam* allele (Law et al., 2003) to several CreER lines that drive recombination in radial glia-like (RGL) stem cells (*Nestin-CreER*), late neuronal progenitors (*Neurod1-CreER*), late neuronal progenitors and immature neurons (*Dcx-CreER*), and mature neurons (*Camk2a-CreER*). For simplicity, mutants are denoted as L1^Nestin^, L1^Neurod1^, L1^Dcx^, and L1^Camk2a^. Note, given that cells in the adult neurogenic lineage comprise only a small fraction of the cells in the dentate gyrus and that L1CAM protein is primarily localized to cell processes, which are intermixed with those from cells not in the neurogenic lineage, it was not possible to confirm loss of L1CAM protein by either immunohisctochemistry or Western blot analysis for the CreER lines used here. Nevertheless, the driver lines used here have been previously validated with multiple floxed alleles (Balordi and Fishell, 2007; Aprea et al., 2014; Madisen et al., 2010).

Due to the X-linked nature of *L1cam*, mutant males were hemizygous and mutant females homozygous for the conditional floxed *L1cam* allele. Mutant males and females were similar in the phenotypic parameters examined and were therefore pooled for analyses. Controls for mutants were littermates that did not carry the respective CreER transgene. All mice, mutants and controls, received tamoxifen (TM), which was used to activate CreER.

**Fig. 1.**
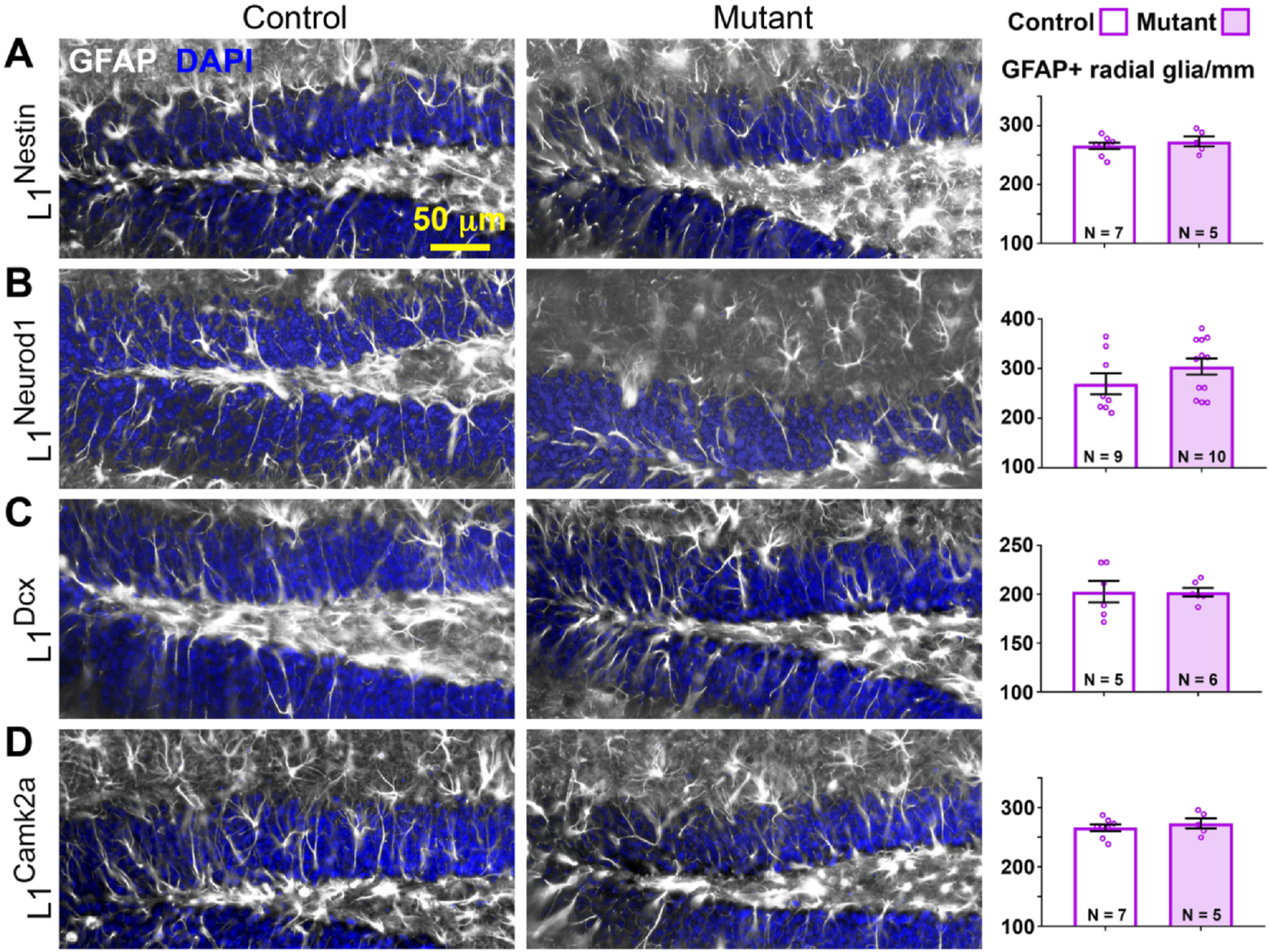
Loss of L1 does not impact numbers of radial glia-like stem cells. Sections of controls and L1^Nestin^ (A), L1^NeuroD1^ (B), L1^DCX^ (C) and L1^Camk2a^ (D) mutants were stained for GFAP (white) and counterstained for DAPI (blue). Scale bar: 50 μm. Quantification of GFAP + cells/mm was performed for the dorsal blade of the DG. N = animal number per genotype. Two-tailed Student’s *t*-test, mean ± SEM.

To determine if L1 is autonomously required in adult RGL stem cells in the DG, RGL stem cell numbers were examined in 2- to 3-month-old control and mutant L1^Nestin^ mice 5 weeks after TM treatment, a time sufficient for slowly dividing stem cells to exhibit a difference in numbers between experimental groups (e.g. Kang and Hébert, 2015). RGL stem cells were defined as GFAP + cell bodies in the subgranular zone (SGZ) with a radial process extending vertically through the granule neuron layer. No significant differences in RGL stem cells were detected (Fig. 1A). As an independent measure of the number of stem cells and early progenitors, the numbers of SOX2 + PCNA + cells in L1^Nestin^ and L1^Dcx^ mutants were examined and found to be similar to their respective controls (Figs. S1A–C, S2). On the other hand, the number of SOX2 + BrdU + cells (where BrdU was administered for 2 days immediately before collecting the brains) was slightly decreased in L1^Nestin^ but not L1^Dcx^ mutants (Fig. S3), consistent with premature differentiation of progenitors. These results suggest that L1 does not autonomously regulate stem cell numbers in the DG, but may be required to curb their proliferative state.

To determine if RGL stem cells are in part regulated by an L1-mediated feedback signal from surrounding cells in the neurogenic lineage, RGL cell numbers were also examined in control and mutant L1^Neurod1^, L1^Dcx^, and L1^Camk2a^ mice 5 weeks after TM treatment. The results showed no differences between control and mutant groups (Fig. 1B–D), suggesting that L1 also does not regulate RGL cell numbers non-autonomously in the DG.

### 2.2. L1cam loss in late progenitors and immature neurons leads to an increase in new neurons

Despite no observed differences in RGL cell numbers, L1 could nevertheless play roles at later steps in the neurogenic lineage. To address this possibility, potential effects on one of the last stages of neurogenesis, the differentiation of new neurons to a NeuN + state, were examined. To determine the numbers of new neurons, BrdU was administered for 7 days immediately after TM treatment to pulse-label newborn cells. Mice were sacrificed 28 days later and the number of new surviving neurons, i.e. those stained for both NeuN and BrdU, was quantified. Virtually all BrdU + cells were also NeuN +.

**Fig. 2.**
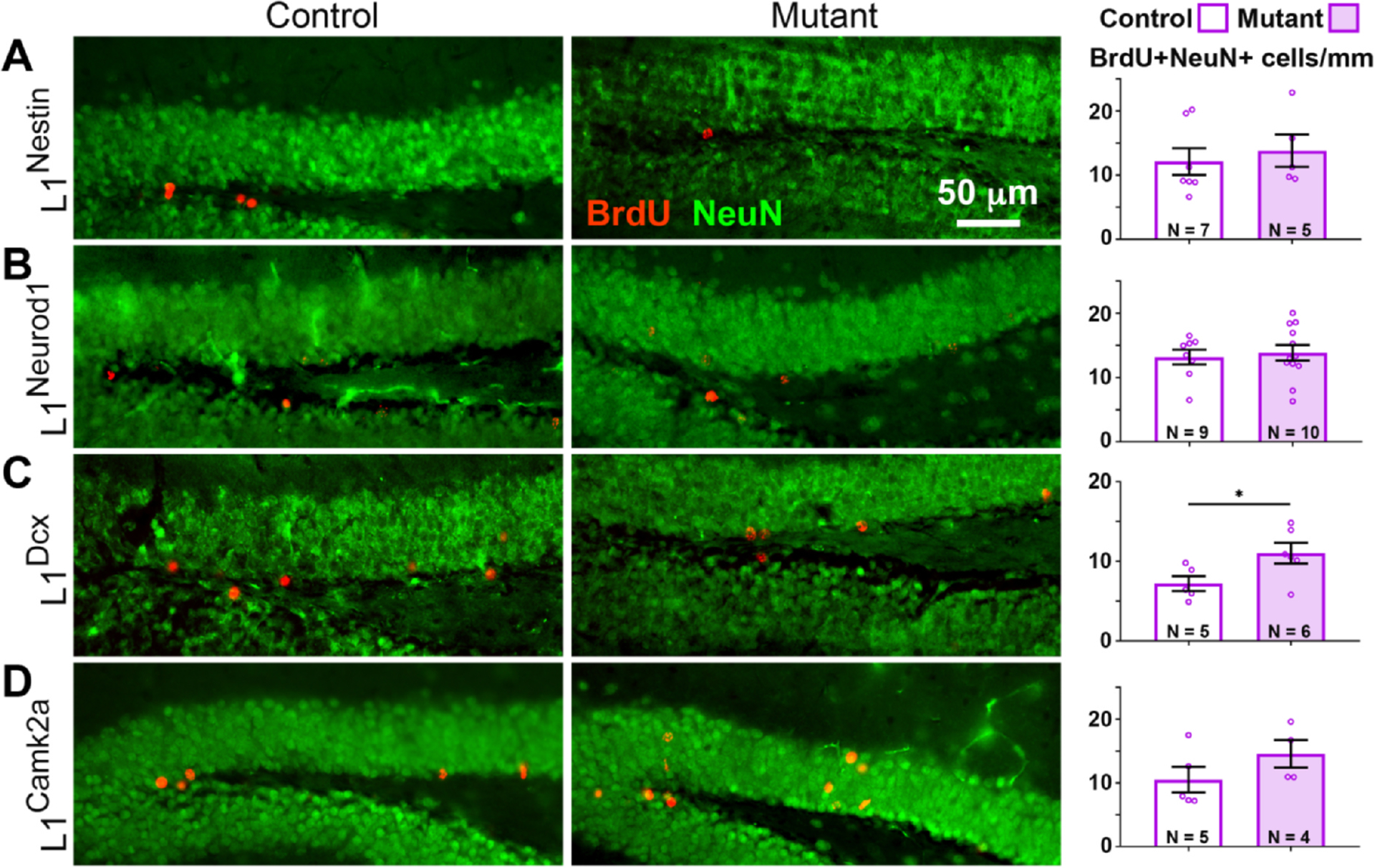
Numbers of new neurons are reduced in L1^DCX^ mutants. New mature neurons were stained for BrdU (red) and NeuN (green) in controls and L1^Nestin^ (A), L1^NeuroD1^ (B), L1^DCX^ (C) and L1^Camk2a^ (D) mutants. Scale bar: 50 μm. Quantification of BrdU + NeuN + cells/mm was performed for the dorsal blade of the DG. N = animal number per genotype. Two-tailed Student’s *t*-test; mean ± SEM; *, p = 0.0485.

L1^Dcx^ mutants compared with controls exhibited a small but significant increase in new neurons (Fig. 2C), which were positioned slightly closer to the SGZ, suggesting that they had not on average migrated as far as in control mice (Fig. S4). In contrast, L1^Neurod1^ and L1^Nestin^ mutants did not show such an increase compared to their respective controls (Fig. 2A, B), possibly due to differences in the efficiency or timing within the differentiation lineage of recombination between the CreER driver lines. To address the possibility that L1^Nestin^ mutants may take longer to exhibit a change in BrdU + NeuN + cell numbers or require a longer TM regimen to more efficiently recombine the *L1cam*^*fx*^ allele, we doubled the number of TM treatments from 5 to 10 and extended the length of time to analysis by 1 week. Although the data trended even more than with the shorter timeline toward an increase in BrdU + NeuN + cells in L1^Nestin^ mutants (consistent with the L1^Dcx^ mutants), the differences did not reach statistical significance (Fig. S1D–F).

L1^Camk2a^ mutants, in which only mature neurons are targeted for recombination, also did not exhibit a difference in the number of new neurons compared to controls (Fig. 2D), consistent with there being no detectable L1-mediated non-autonomous effects from mature neurons on neurogenesis. Note that the number of BrdU + NeuN + cells in control mice for each Cre group is different, particularly for the L1^Dcx^ where the numbers are lower, exemplifying the importance of using littermate controls for each of these background strains.

### 2.3. L1cam loss results in fewer progenitor cells

The observed increase in the numbers of new neurons in L1^Dcx^ mutants could be explained through one of two mechanisms. First, more new neurons could reflect an increase in the number of progenitors. Alternatively, and as observed in neurodevelopmental contexts involving FGF signaling (Kang et al., 2009), an increase in mature neurons shortly after the deletion of L1 could reflect premature differentiation of progenitors that leads to an initial, but not long term, increase in neurons.

**Fig. 3.**
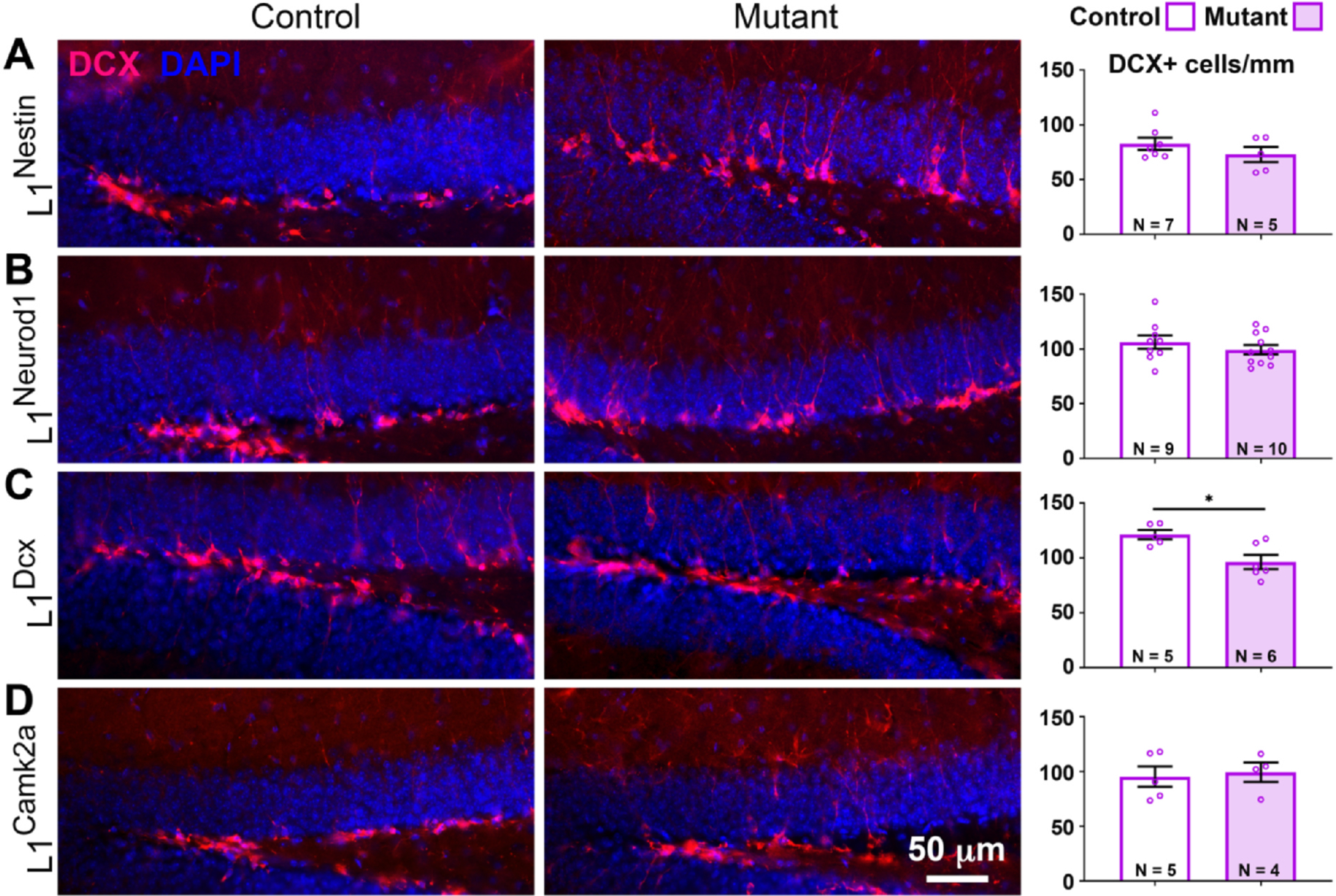
Numbers of late progenitors and immature neurons are increased in L1^DCX^ mutants. Late progenitors and immature neurons were stained for DCX (red) and counterstained with DAPI (blue) in controls and L1^Nestin^ (A), L1^NeuroD1^ (B), L1^DCX^ (C) and L1^Camk2a^ (D) mutants. Quantification of DCX + cells/mm was performed for the dorsal blade of the DG. N = animal number per genotype. Scale bar: 50 μm. Two-tailed Student’s *t*-test; mean ± SEM; *, p = 0.0131.

To address whether the increase in new neurons was the result of increased numbers of progenitors or to premature progenitor differentiation, the number of DCX + cells (late progenitors and immature neurons) was determined. L1^Dcx^ mutants displayed fewer DCX + cells than controls (Fig. 3C), suggesting that loss of L1 causes progenitors to prematurely differentiate rather than increase their numbers. Consistent with the observation that numbers of mature neurons in L1^Nestin^, L1^Neurod1^, and L1^Camk2a^ mutants were similar (Fig. 2), the number of DCX + cells in these mutants compared to controls was not detectably different (Fig. 3A, B, D).

### 2.4. Pan-neuronal loss of L1cam throughout development and adulthood leads to an earlier decline in neurogenesis with age

Although non-autonomous effects were not detected when deleting *L1cam* from mature neurons in adult mice (Figs. 1D, 2D, 3D), it is possible that due to perdurance of L1 protein, an insufficient amount of L1 was reduced as a result of gene deletion. To test this possibility, we deleted the floxed *L1cam* allele with *Synapsin*^*Cre*^. *Synapsin*^*Cre*^ drives recombination in differentiated neurons as soon as they appear in the brain by E12.5 (Zhu et al., 2001). As a result, we observed extensive loss of L1 protein by Western blot analysis of hippocampi from adult *Synapsin*^*Cre*^*;L1cam*^*fx*^ (L1^Synapsin^) mice (Fig. S5). RGL stem cells and progenitors were examined in the DG of L1^Synapsin^ mutants and controls from 3 to 12 months of age, a period during which adult neurogenesis sharply declines (Kang and Hébert, 2015).

**Fig. 4.**
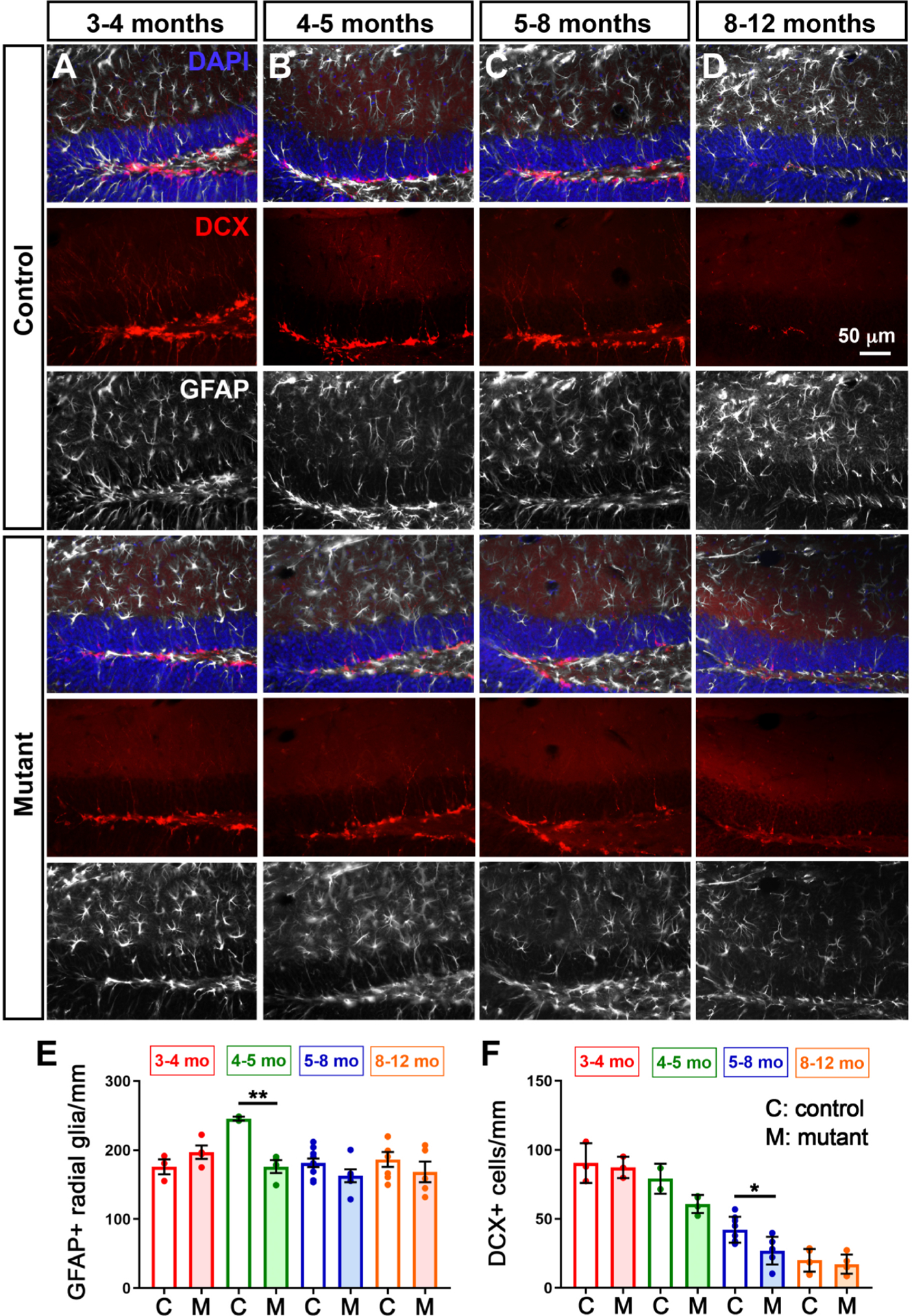
Loss of L1 in neurons throughout development and adulthood eventually reduces adult neural precursors in the DG. Immature neurons were stained for DCX (red), RGL stem cells for GFAP (white), and nuclei with DAPI (blue) at different stages of adulthood: 3–4 months (A); 4–5 months (B); 5–8 months (C); and 8–12 months (D). (E) Quantification of GFAP + radial glia/mm. Two-way ANOVA: interactions: age, *p* = 0.0192; genotype, *p* = 0.0136, with Sidak posthoc tests. **p = 0.0066. (F) Quantification of DCX + cells/mm. Two-way ANOVA: interactions: age, *p* < 0.0001; genotype, *p* = 0.0048, with Sidak posthoc tests. *p = 0.0116. Mean ± SEM.

The expected decline with age in neurogenesis occurred similarly in mutants and controls, with two differences (Fig. 4). First, the decline in GFAP + RGL stem cells and in DCX + late progenitors/early neurons was more prominent in mutants than controls, particularly at 4–5 months of age for stem cells and 5–8 months of age for progenitors (Fig. 4A–F). These results suggest that over extended periods of time L1 expressed by neurons can play a non-autonomous role in maintaining normal levels of neurogenesis. These data also suggest that with age, *L1cam* deficiencies could reduce DG-based cognition, particularly spatial pattern separation (Clelland et al., 2009), and cause inappropriate stress responses (Hill et al., 2015).

### 2.5. Deletion of L1cam in the neurogenic lineage increases dendrite complexity

**Fig. 5.**
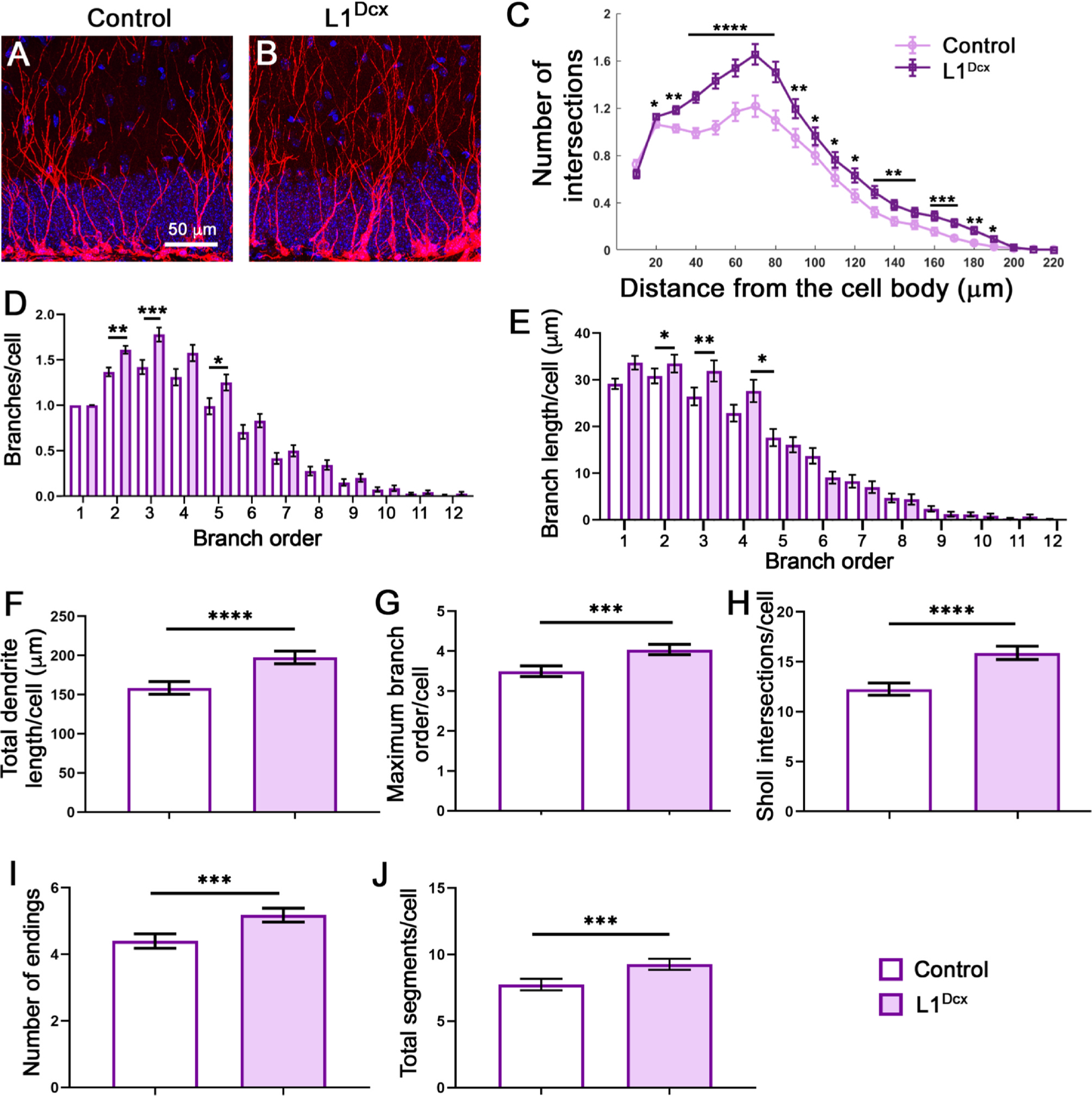
Deletion of *L1cam* results in dendrites with increased branching. Sections of control (A) and mutants (B) were immunostained for DCX (red) and DAPI (blue). (C) Sholl analysis was performed showing the number of intersections over the distance from the cell body. p values for significant differences are: 20 μm, 0.0347; 30 μm, 0.0014; 40–80 μm, < 0.0001; 90 μm, 0.0041; 100 μm, 0.0310; 110 μm, 0.0192, 120 μm, 0.0132; 130 μm, 0.0047; 140–150 μm, 0.0024; 160 μm, 0.0006; 170 μm, 0.0008; 180 μm, 0.0039; 190 μm, 0.0166. N = 5 mice and n = 370 cells for controls; N = 5 mice and 402 cells for mutants. Two-tailed Mann Whitney test; mean ± SEM. (D) Number of branches by branch order per cell. p = 0.0267; **, p = 0089; ***, p = 0.0008. (E) Branch length by branch order per cell. *, p = 0.0494 (2° branch); **, p = 0.0019 (3° branch); *, p = 0.0111 (5° branch. The number of mice and cells analyzed in (D) and (E) are as in (C). For (D) and (E), a one-way ANOVA was performed, mean ± SEM. (F) Total dendrite length per cell. ****, p < 0.0001; N = 5 mice and 375 cells for controls and 5 mice and 400 cells for mutants. (G) Maximum branch order per cell. ***, p = 0.0007. Number of mice and cells are as in (E). (H) Sum of Sholl intersections per cell. ****, p < 0.0001. N = 5 mice and 370 cells for controls and 5 mice and 402 cells for mutants. (I) The total number of terminal endings per cell between controls and mutants. ***, p = 0.0005. (J) The total number of branch segments per cell between controls and mutants. ***, p = 0.005. N = 5 mice and 375 cells for control and 5 mice and 400 cells for mutants in (I,J). (F-J) Two-tailed Mann-Whitney test; mean ± SEM.

L1 has been shown to be required for proper neurite elaboration across species. To assess whether *L1cam* is also required autonomously within the neurogenic lineage for dendrite elaboration in the adult DG, dendrites in L1^Dcx^ and L1^Nestin^ mutants were compared to their respective controls. Dendrites were examined by Sholl analysis ten days (L1^Dcx^) or two weeks (L1^Nestin^) after TM treatment, a time when newborn neurons derived from recombined stem/progenitor cells would be growing and remodeling dendrites, a process required for proper circuit integration and survival (Gonçalves et al., 2016). Immature neurons were identified by DCX staining and 3D reconstructions of individual neurons were performed using confocal imaging and Neurolucida software (Fig. 5A, B).

Unexpectedly, the dendritic trees in mutants displayed increased elaboration compared with controls, particularly in the L1^Dcx^ mutants (Fig. 5), but also to a lesser but still significant extent in the L1^Nestin^ mutants (Supplemental Fig. 6). Both the L1^Dcx^ and L1^Nestin^ mutants compared with their respective controls exhibited more complex dendritic trees by Sholl analysis with greater branching, branch lengths, total dendrite lengths, higher maximal branch orders, number of endings, and number of segments (Fig. 5, Supplemental Fig. 6). The distribution of cells for each of these quantifications is provided (Supplemental Figs. 7 and 8).

### 2.6. Deletion of L1cam increases anxiogenic-related behavior

Given the cellular phenotypes in the DG described above, deletion of *L1cam* in progenitors and newborn neurons could impact hippo-campus-regulated behaviors such as anxiety. To test this possibility, we examined the behavior of control and L1^Dcx^ mutants and controls in the elevated plus maze 1 month after TM treatment. In the elevated plus maze, two of the opposing arms are open (i.e. with no side walls) and the remaining two arms are closed (with side walls). Mice have a tendency to avoid open spaces but are also prone to exploratory behavior of new environments. Mice exhibiting anxiety-like behaviors tend to spend more time in the closed arms of the maze, as opposed to open arms.

**Fig. 6.**
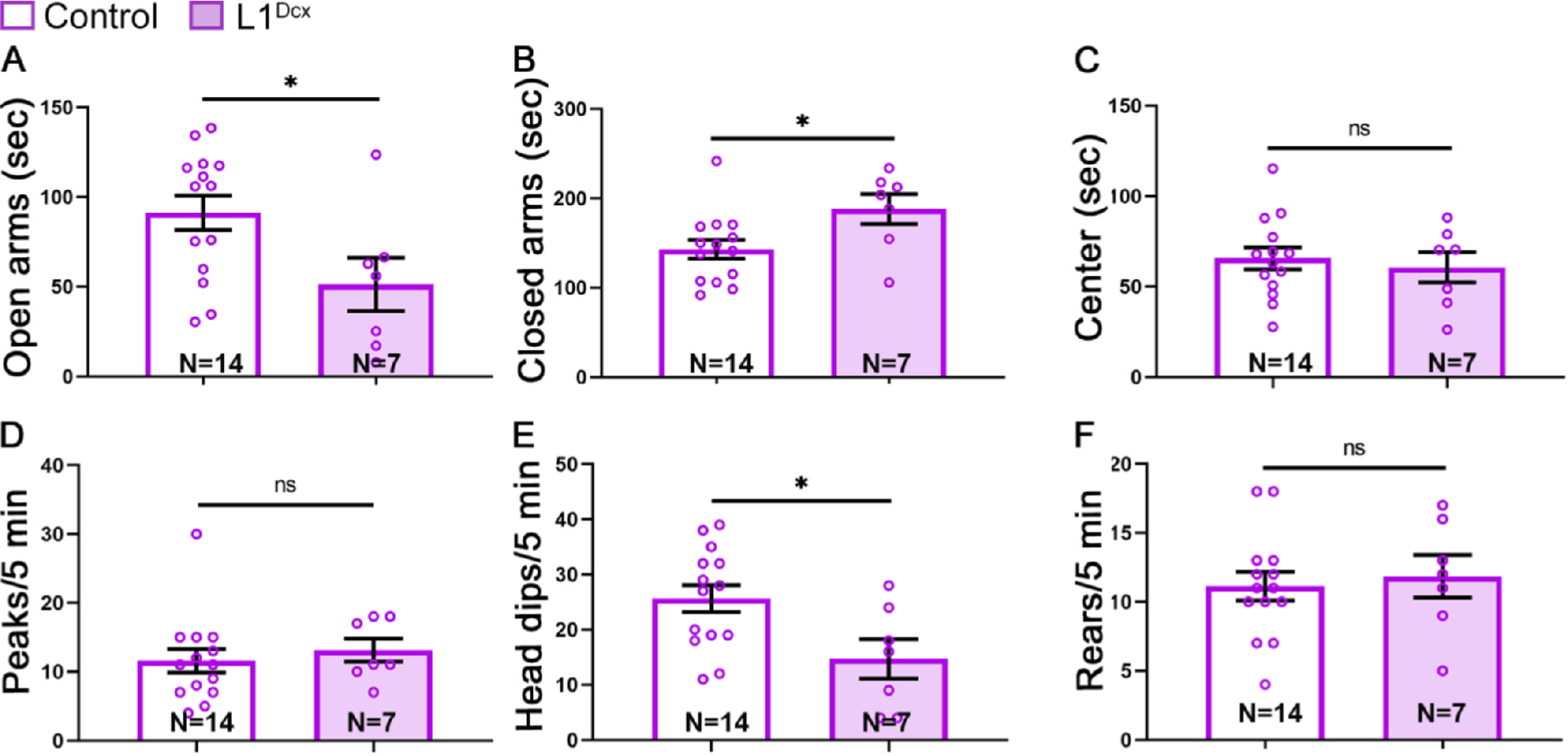
Anxiogenic-like behavior is increased in in L1^DCX^ mutants. Mice were tested on the elevated plus maze 4 weeks post-TM. (A) Time spent in open arms. *, p = 0.0316. (B) Time spent in closed arms. *, p = 0.0285. (C) Time spent in the center of the maze. (D) Number of head peaks in to the open arms. (E) Number of head dips underneath the open arms. *, p = 0.0198. (F) Number of rears in the closed arms. N = number of animals. Two-tailed Student’s *t*-test; mean ± SEM.

Mutant mice spent less time in the open arms and more time in the closed arms compared with control mice (Fig. 6A, B). Time spent in the center of the plus maze (Fig. 6C), the number of times a mouse peeked from out of the closed arms into the open arms (Fig. 6D), and the number of times a mouse reared (Fig. 6F) were not detectably different in both groups. As an additional measure of anxiety, mutant mice dipped their heads under the open arms fewer times than control mice (Fig. 6E). Together, these results suggest that loss of L1 in late progenitors and immature neurons of the DG results in increased anxiety.

## 3. Discussion

In this study, we identified requirements for L1 in adult hippo-campal neurogenesis. First, deletion of *L1cam* in neuronal progenitors led to an increased number of mature neurons coincident with a decreased number of progenitors, suggesting that L1 curbs the premature differentiation of progenitors. Second, deletion of *L1cam* resulted in more branching and extension of dendrites in immature DCX + neurons, suggesting that L1 suppresses dendrite elaboration. Third, deletion of *L1cam* in late progenitors and immature neurons led to increased anxiogenic behaviors in the elevated plus maze assay, suggesting that L1 deficiency could lead to increased anxiety.

Noteworthy is the extent to which the phenotypes differed between L1^Nestin^, L1^Neurod1^, and L1^Dcx^ mutants. In terms of changes in cell numbers, while L1^Nestin^ and L1^Dcx^ mutants showed the same trends, L1^Neurod1^ mutants, surprisingly, had no detectable phenotype. Given the differing baseline levels of neurogenic cells in the control mice for each experimental group, we attribute the lack of phenotype in L1^Neurod1^ mutants at least in part to strain differences, but we cannot exclude the possibility that the *Neurod1-CreER* driver did not sufficiently delete the floxed *L1cam* allele.

In addition, the L1^Nestin^ mutants were also expected to encompass all the phenotypes of the L1^Dcx^ mutants, but instead showed a milder phenotype both in terms of cell numbers and dendrite complexity. Even when using an extended experimental timeline after tamoxifen treatment, cell numbers for the most part were not significantly different from controls in L1^Nestin^ mutants (except for a decrease in SOX2 + BrdU + cells). It is possible that the extended timeline was still too short to detect a more robust phenotype. However, this seems unlikely due to phenotypes observed when other floxed genes are deleted within shorter time frames using the same *Nestin-CreER* driver (e.g. Balordi and Fishell, 2007; Kang and Hébert, 2015).

There are at least three other possibilities for why L1^Nestin^ mutants showed a milder phenotype than L1^Dcx^ mutants. One, recombination may be less efficient (i.e. a smaller fraction of neurogenic cells become recombined in L1^Nestin^ versus L1^Dcx^ mutants). However, even doubling the TM treatment in L1^Nestin^ mutants did not greatly impact the phenotype. And this Nestin-CreER driver can efficiently recombine other alleles (e.g. Kang and Hébert, 2015). Hence, we do not favor this as the main explanation. Another possibility is that stem cells deficient in L1 were selected against due to a requirement for L1 in maintaining these cells. Although this possibility cannot be excluded, the clear dendritic phenotype of young neurons in these mutants indicates that at least some recombined cells progress beyond the progenitor stages of differentiation. Finally, as with the L1^Neurod1^ mutants, differences in strain background could account for the differing severities in the phenotypes of L1^Nestin^ and L1^Dcx^ mutants.

L1-mediated suppression of dendrite elaboration was unexpected given that L1 in other assays promotes axon and dendrite length and branching (Diaz-Balzac et al., 2015; Forni et al., 2004; Doherty and Walsh, 1996; Saffell et al., 1997; Patzke et al., 2016; Salzberg et al., 2013; Dong et al., 2013; Zou et al., 2016). However, these findings are potentially reconcilable if one considers that in both cases L1 can act as an adherence guidance cue. In some contexts, neurites deficient in L1 might not recognize their target and stall, while in other contexts such as the DG projections untethered by L1 might continue extending exuberantly beyond their normal targets. Alternative explanations are possible. For instance, in the DG, L1 may simply act as an adhesive substrate to restrain dendrite extension whereas in other cases it may act as a ligand to regulate receptor kinase-induced process extension (e.g. Castellani et al., 2000).

The effects described here for loss of *L1cam* could be considered modest. This could be due to compensation by other Ig-containing proteins. In particular, *L1cam*’s other subfamily members (*Chl1*, *Nrcam*, *Nfasc*), with which *L1cam* shares ~40% homology, are also expressed in the adult DG (Allen Brain Atlas). In addition, many other genes of the Ig-containing superfamily are also expressed in the DG. Thus, despite the many Ig-containing proteins expressed in this brain area, it is in some respect remarkable that a phenotype is detected with loss of *L1cam* alone, underscoring its potential importance in hippocampal neurogenesis.

We found that loss of *L1cam* in stem/progenitor cells of the adult brain also resulted in an increase in anxiety-related behavior. Whether these changes in behavior in *L1cam* mutants are due directly to the changes in cell differentiation and dendrite elaboration in the DG remains uncertain because of the potential for behavior-altering defects in other neurogenic niches. First, the *Dcx* driver line used in this study also recombines lox alleles in neuroblasts derived from the anterior subventricular zone, which generates neurons destined for the olfactory bulb. Olfactory bulbs were not analyzed in this study but defects within them could in theory impact the behavior of the mice in the elevated plus maze. Second, loss of *L1cam* in new immature neurons could non-autonomously affect synaptic transmission of existing mature neurons, as described in certain *C. elegans* neurons (Opperman et al., 2015), thus indirectly impacting behavior. Finally, the observed behavioral difference could alternatively be due to disruption of amygdala neurons since these also express DCX (Jhaveri et al., 2018).

Importantly, the observed increase in anxiety-related behavior in L1 mutants was unexpected given that increases in hippocampal neurogenesis have been shown to reduce, rather than increase, anxiety (e.g. Hill et al., 2015). Therefore, the increase in differentiated neurons and in the elaboration of dendrites described here must differ mechanistically from the increases reported previously. Similarly, if L1 were acting as a ligand for FGFRs or TRKs, both of which promote dendrite elaboration in newborn DG neurons (Bergami et al., 2008; Kang and Hébert, 2015), then L1 would need to interact with these receptors in as yet unidentified ways that interfered with their pro-neurogenic and prodendritogenic activity. Future experiments are needed to address the mechanism of action of L1. However, regardless of the mechanisms by which L1 regulates neuron production, dendrite elaboration, and increased anxiety, the results provided here demonstrate the importance of L1 to different aspects of adult hippocampal neurogenesis.

## 4. Experimental procedures

### 4.1. Mice and tamoxifen administration

All experiments were approved by the Albert Einstein College of Medicine Institutional Animal Care and Use Committee. The following Cre alleles *Nestin-CreER* (Balordi and Fishell, 2007), *Dcx-CreER* (MMRRC:032780, contributed by Ulrich Müller), *NeuroD1-CreER* (Aprea et al., 2014), and *CamKII-CreER* (Madisen et al., 2010) were crossed to a conditional floxed allele of *L1cam*^*fx*^ (Law et al., 2003) to generate homozygous (female) and hemizygous (male) mutants and littermate controls. CreER lines are all CreERT2. Alleles are on mixed strain backgrounds. Two-month-old mutants and *Cre*-negative control littermates were administered an intraperitoneal injection of tamoxifen (TM) (5 mg/35 g of body weight) dissolved in corn oil, once a day, every other day for a total of 5 doses. Mice were perfused with 4% paraformaldehyde 2 weeks (dendrite analysis) or 5 weeks (cell numbers quantification) post-tamoxifen.

### 4.2. Immunohistofluorescent staining

Vibratome sections (Leica VT1000S) (30 μm) were washed 3 times in PBS, blocked in 10% normal goat serum, 1% bovine serum albumin, 100 mM glycine, 0.1% Triton-X, in PBS for 1 h at room temperature and incubated with primary antibodies overnight at 4 °C in 1% normal goat serum, 1% bovine serum albumin, 0.1% Triton-X, in PBS. Samples were then washed in PBS and incubated in secondary antibodies for 1 h at room temperature. Primary antibodies used were: guinea pig anti-DCX (1:2000, Millipore, AB2253), rabbit anti-GFAP (1:1000, Daco, Z0334), rat anti-BrdU (1:1000, Bio-Rad, OBT0030G), mouse anti-L1cam (1:300, Abcam, [2C2] ab24345), rabbit anti-Sox2, (Millipore, ab5603) (1:2000), mouse anti-PCNA (Abcam, ab29) (1:500). PCNA was detected with antigen retrieval in sodium citrate buffer (10 mM Sodium Citrate, 0.05% Tween 20, pH 6.0) at 95 °C hot plate for 45 min, cooled down for 20 min, and washed 2x in PBS for 5 min. Secondary antibodies used were goat anti-mouse, -rabbit, -guinea pig, and -rat Alexa Fluor 488, 568, and 647. For BrdU staining, before the blocking step, sections were incubated in 2 M HCl at 37 °C for 30 min, followed by 0.1 M sodium borate, pH 8.5 for 20 min, and PBS. Sections were imaged using a Zeiss AxioSkop2 or a confocal Zeiss LSM880 Airyscan Confocal Microscope.

### 4.3. BrdU administration

Newly born adult neurons were labeled by injecting 2-month old mice intraperitonially with BrdU (100 mg/kg body weight) dissolved in 0.9% saline 2x/day for 7 days, starting a day after the last TM administration.

### 4.4. Cell counts

A minimum of five 30 μm sections centered on Bregma −1.34 180 μm apart were used to count cells along 450 μm of the dorsal blade in 4–10 mice for each experimental group, except for Fig. 4, where 3–7 mice were examined per group. Cells were counted in the dorsal blade of positionally-matched sections between animals (blind to genotypic group). Counts were normalized to length, as labeled in the figures. All cells within a field were included in the analyses (no exclusions).

### 4.5. Dendrite measurements

Mice were perfused 2 weeks after the last administration of TM. Thirty μm sections (which were at the same relative position as those used for cell counts) were stained for DCX, imaged using a confocal Zeiss LSM880 Airyscan Confocal Microscope with 40x C-Apochromat/1.2 W Korr FCS M27, and confocal 3D images (optical section = 0.1 um) were traced using a Neurolucida 360 software. All cells within a field were included in the analyses (no exclusions). Experimenter was blind to genotypes. All data was exported to Excel and analyzed using GraphPad Prism 8.1.2. Sholl analysis was graphed using MATLABR2018b.

### 4.6. Anxiety-related behaviors

The elevated plus maze was performed to measure anxiety-related behavior. Mice were housed in a 12-h light/dark cycle. Male and female mice were transferred to the experimental room for 1 h before the test for acclimatization. The maze was comprised of two perpendicular arms: open arm (25 cm long × 5 cm wide × 0.5 cm walls), closed arm (25 cm long × 5 cm wide × 15 cm walls), and a center platform (5 × 5 cm). The apparatus was positioned 50 cm from the floor, on a white background. The maze was illuminated from above. Mice were lowered into the maze facing the closed arm. Each mouse was allowed to freely explore the maze for 5 min. The mice were video-recorded with a camera positioned above the maze. Scoring by the experimenter was done blind to the genotype. Number of entries into each arm, time spent in each arm, and numbers of rearings, head dips under the open arm, and risk assessments were scored. A mouse was considered in a zone when all four paws were touching that zone. Animals were tested only once.

### 4.7. Western blotting

Brain tissue was lysed with RIPA buffer with Proteinase inhibitors (P8340–5 ml Sigma), sonicated for 2 cycles of 10-s on a 30% power using a sonicator (Misonix S4000 with a microtip on the ultrasonic convertor). Debris was spinned at 14,000 rpm for 10 min at 4 °C. Soluble protein concentration was quantified using BCA Protein Assay (ThermoFisher Scientific Cat. 23227) according to manufacturer’s recommendations, imaged using (Machine name), and standard curve was generated using Omega Software. Samples were heated for 5 min at 95 °C in 1X Laemmli sample buffer (375 mM Tris.HCl, 9% SDS, 50% Glycerol, 0.03% Bromophenol blue, 0.6 M DTT). 20 μg of protein samples were loaded onto Mini-PROEAN TGX (4%–20%) (Bio-Rad, Cat. 456–1094), run using Tris-Glycine-SDS (0.25 M Tris Base, 1.92 M Glycine, and 1.0% (w/v) SDS) for sodium dodecyl sulfate-gel electrophoresis (SDS-PAGE) and transferred onto 0.4 μm PVDF membrane (GE AMersham, FIsherScientific, Cat: 45004110), activated in 100% methanol, using Mini Trans-Blot Transfer System (Bio-Rad Mississauga, ON, CA) for 2 h at 50 V. Membranes were blocked in 5% milk in TBST. Primary antibodies were incubated in Licor TBS block Odyssey® Blocking Buffer in TBS 0.1% sodium azide (Cat. 927–50000) overnight at 4 °C. Primary antibodies used mouse anti-L1cam [2C2] (Abcam, Cat. ab243345) at 1:1000 dilution and rabbit beta-tubulin (D2N5G) (Cell Signaling, Cat. 15115) at 1:1000 dilution. Secondary antibodies were diluted 1:10,000 and incubated for 2 h at RT. Secondary antibodies used: IRDye 800CW goat anti-mouse (LI-COR, Cat. 827–08364) and IRDye 680RD goat anti-rabbit (LI-COR, Cat. 926–68171). Fluorescence was detected using Odyssey CLx Infrared Imaging System (Li-Cor Bioscience, Lincoln, NE, USA) according to manufacturer’s recommendations and settings and quantified using Image Studio Lite Software Ver 5.2.

### 4.8. Quantitation and statistics

Statistical analyses were performed with GraphPad Prism 8.1.2 using the two-tailed Student’s *t*-test with *P*-value < 0.5. Dendrite data was analyzed using one-way ANOVA, using Kruska-Wallis test with Dunn’s multiple comparisons test, *P* < 0.05, or two-tailed Mann-Whitney *U* test, *P*-value < 0.5. Synapsin-Cre analysis across different time point was analyzed using two-way ANOVA, with Sidak posthoc tests, *P* < 0.5. Data are represented as mean ± SEM. At least 4 animals were used per group for each experiment.

## Supplementary Material

1
