# Supplementary material for: L1cam curbs the differentiation of adult-born hippocampal neurons
Source: Stem Cell Res. Author manuscript; Available in PMC 2020 Oct 22. (PMC7578921; doi:10.1016/j.scr.2020.101999)
Supplement: 1 [file NIHMS1638444-supplement-1.pdf]

**Supplementary Material**

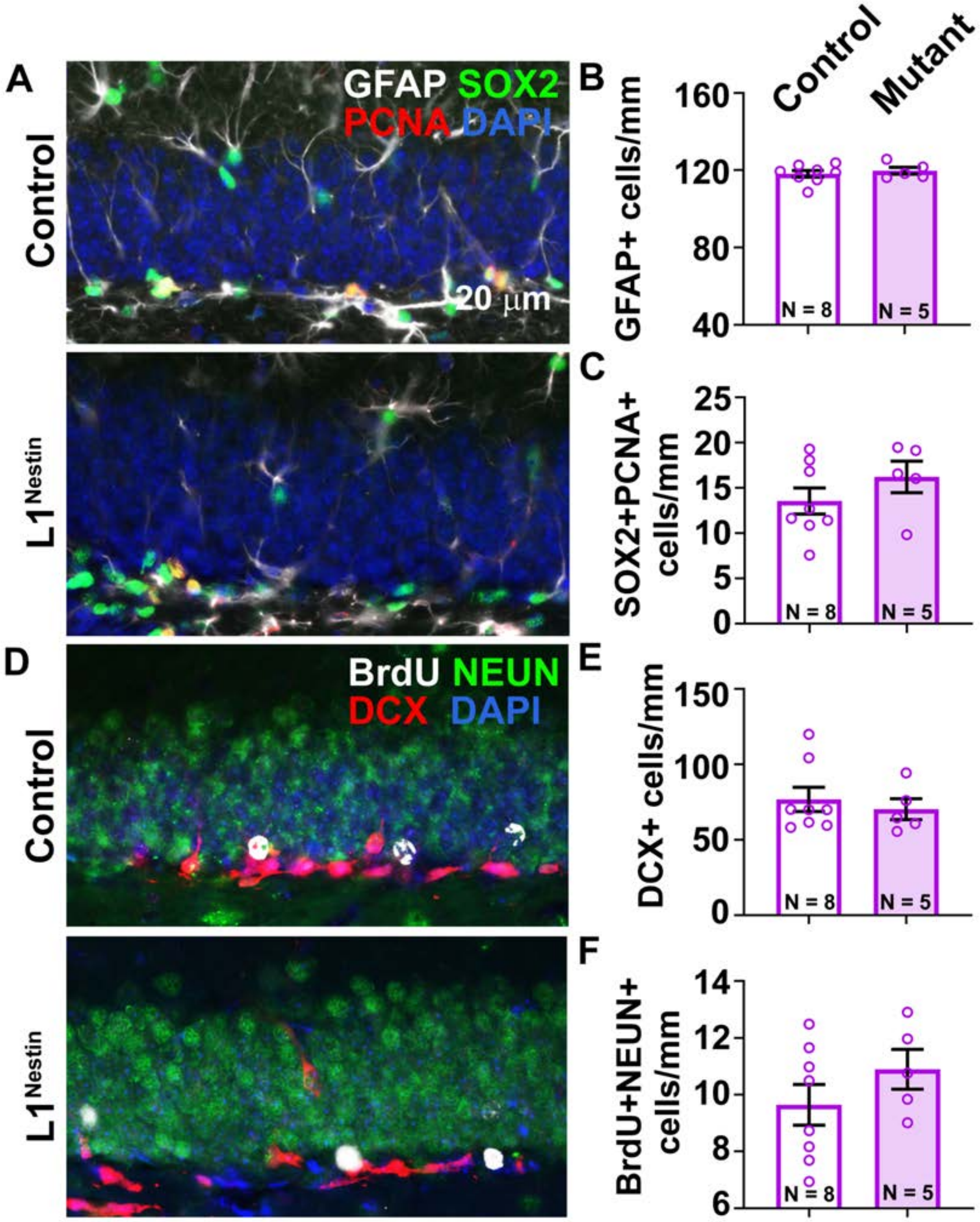


**Figure S1. Quantitation of SOX2+ precursors and BrdU+NEUN+ neurons in L1$^{Nestin}$ mutants. Related to Figure 1**. **(A)** Sections of controls and L1$^{Nestin}$ mutants stained with PCNA (red), SOX2 (green), GFAP (white) and counterstained for DAPI (blue). **(B)** Quantification of GFAP+ cells/mm. **(C)** Quantification of SOX2+PCNA+ cells/mm. **(D)** Sections of controls and L1$^{Nestin}$ mutants stained with DCX (red), NEUN (green), BrdU (white) and counterstained for DAPI (blue). **(E)** Quantification of DCX+ cells/mm. **(F)** Quantification of BrdU+NEUN+ cells/mm. Quantification was performed for the dorsal blade of the

DG 1 month after tamoxifen with an additional 5 days of tamoxifen injections. N, animal number per genotype. Scale bar: 20 µm. Two-tailed Student's t-test; mean ± SEM.

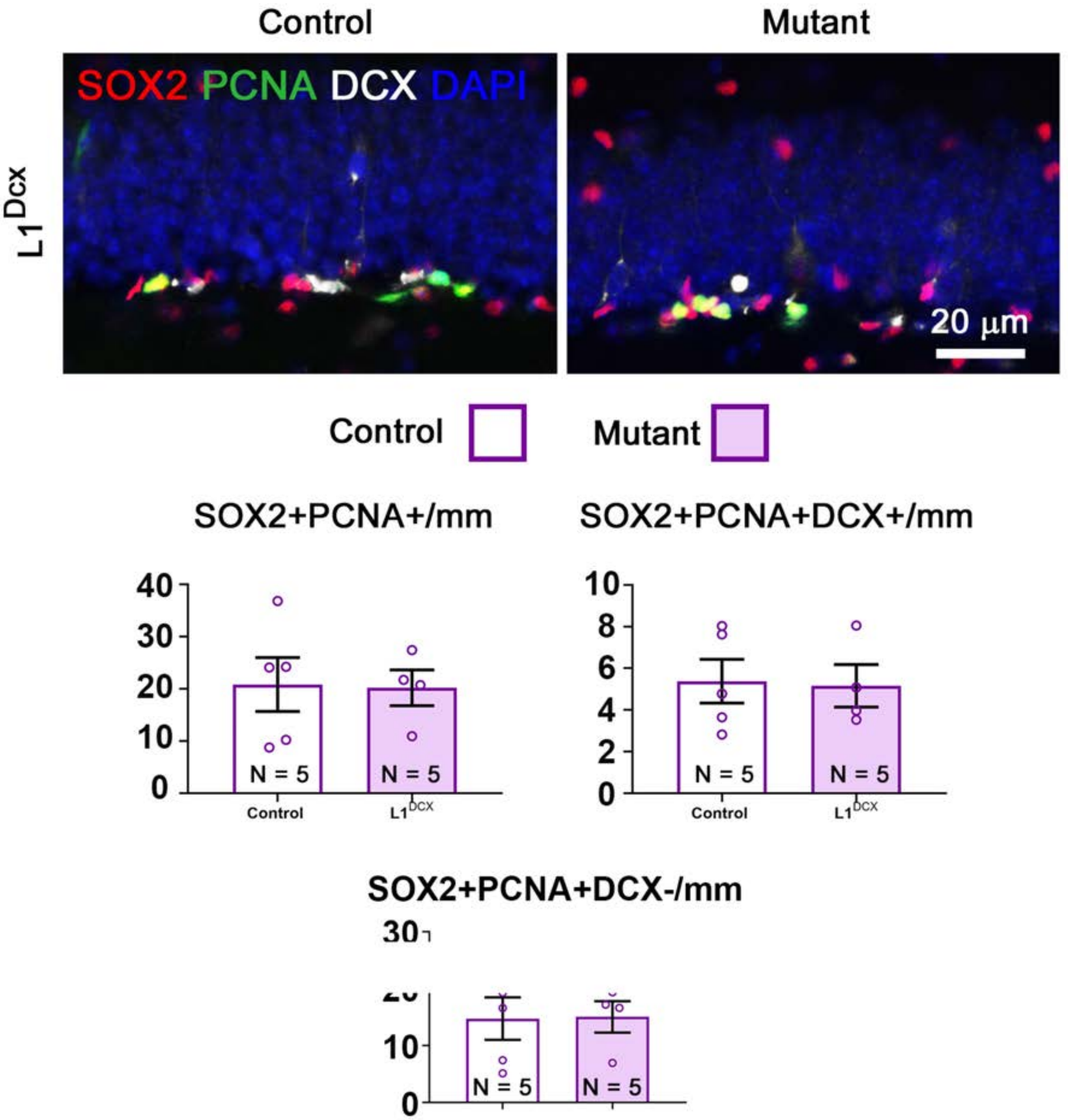


**Figure S2. Quantitation of SOX2+ precursors in L1$^{Dcx}$ mutants. Related to Figure 1**. Sections of L1$^{DCX}$ control and mutant DGs stained with SOX2 (red), PCNA (green), DCX (white) and counterstained for DAPI (blue). Quantification of SOX2+PCNA+ cells/mm, SOX2+DCX+PCNA+ cells/mm, and SOX2+DCX-PCNA+ cells/mm was performed for the dorsal blade of the DG 1 month after recombination. N = 5, animal number per genotype. Scale bar: 50 µm. Two-tailed Student's t-test; mean ± SEM.

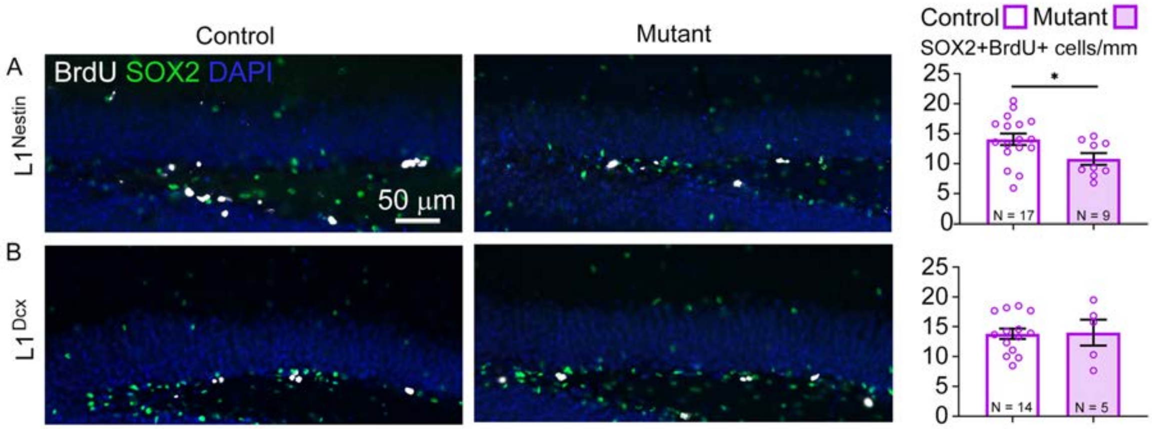


**Figure S3. Quantitation of SOX2+BrdU+ precursors neurons in L1$^{Nestin}$ mutants. Related to Figure 1**. Mice treated with tamoxifen were administered BrdU for 2 days immediately before collecting their brains. Early progenitors were stained for SOX2 (green) and BrdU (white) in L1$^{Nestin}$ **(A)** and L1$^{Dcx}$ **(B)** mutants and their respective controls. Scale bar: 50 µm. Quantification of Sox2+BrdU+ cells/mm was performed for the dorsal blade of the DG. N = animal number per genotype. Two-tailed Student's t-test; mean ± SEM; *$p$ = 0.0394.

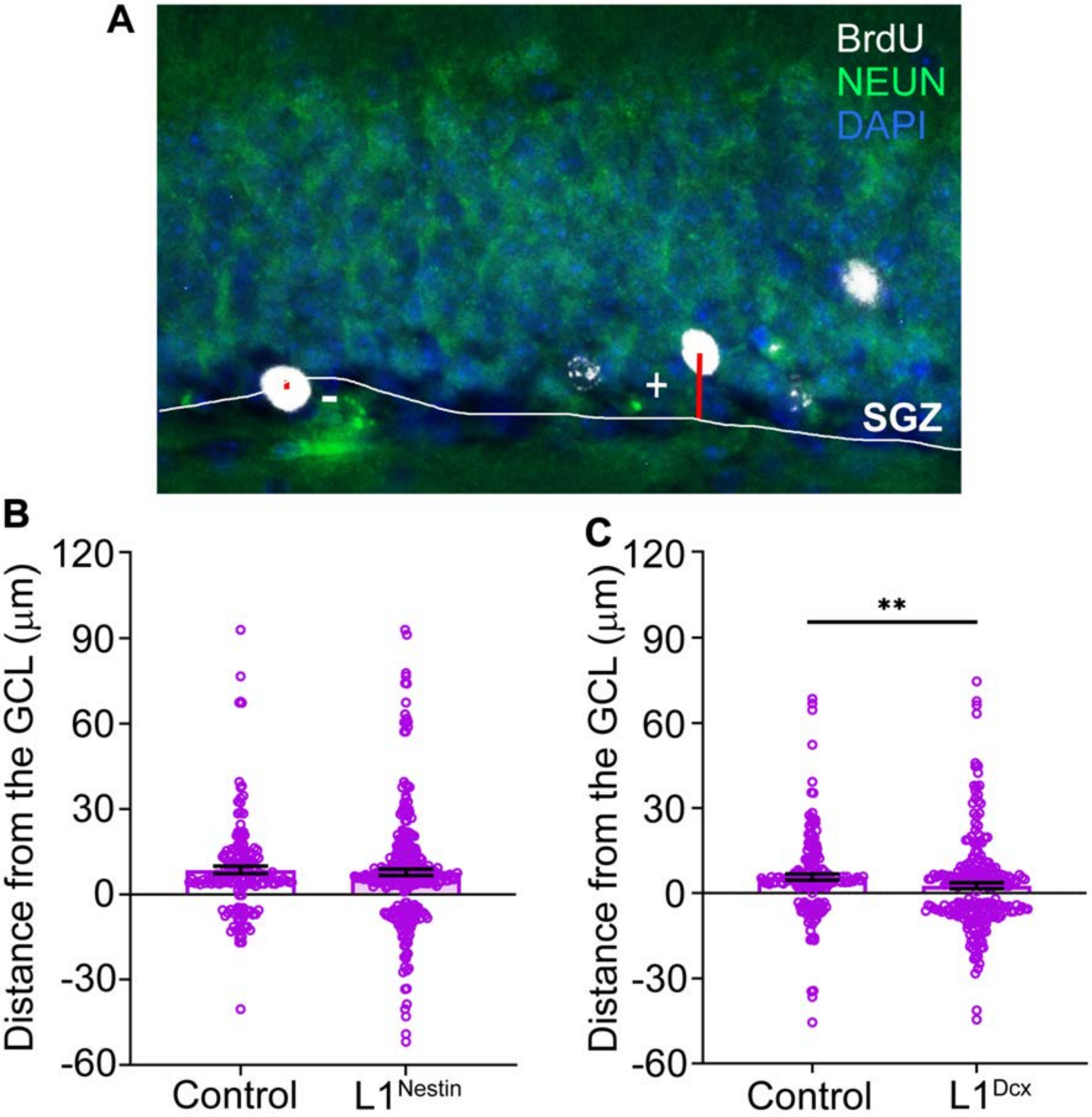


**Figure S4. Distance migrated by BrdU+ cells in the granule layer of mutants. Related to Figure 2. (A)** Example illustrations of distances from the center of a BrdU+ nucleus to the SGZ border; “+” distances are above the SGZ line, while “-“ distances re below. **(B-C)** Quantification of BrdU+NeuN+ cells from the GCL for **(B)** $L1^{Nestin}$ (Control: N = 3, n = 122; and $L1^{Nestin}$: N = 4, n = 256), **(C)** $L1^{Dcx}$ (Control: N = 4, n = 177; and $L1^{Nestin}$: N = 4, n = 237), where N = number of animals, and n = number of cells. Quantification was performed for the dorsal blade of the DG 1 month after recombination. Mann-Whitney test; mean ± SEM; ***p* = 0.0015.

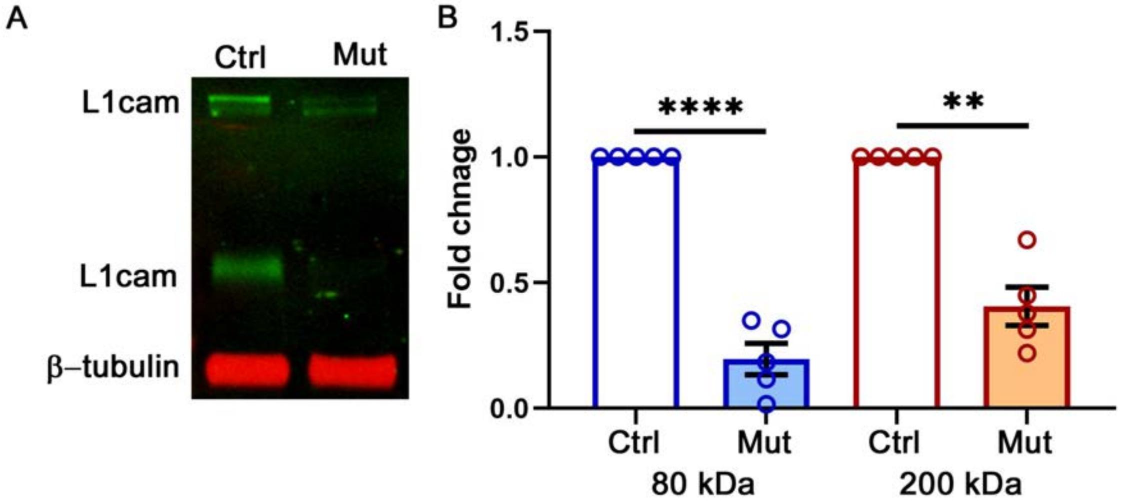


**Figure S5. Loss of L1 protein in L1$^{Synapsin}$ mutants. Related to Figure 4. (A)** Western blot showing reduced L1cam in Synapsin-Cre;L1cam$^{fx/fx}$ (Mut) hippocampi from 4-5 month-old mice compared with controls (Ctrl). **(B)** Quantification of the fold change. N = 5. **** $p$ = 0.0006, ** $p$ = 0.003. One-sample t-test, mean ± SEM.

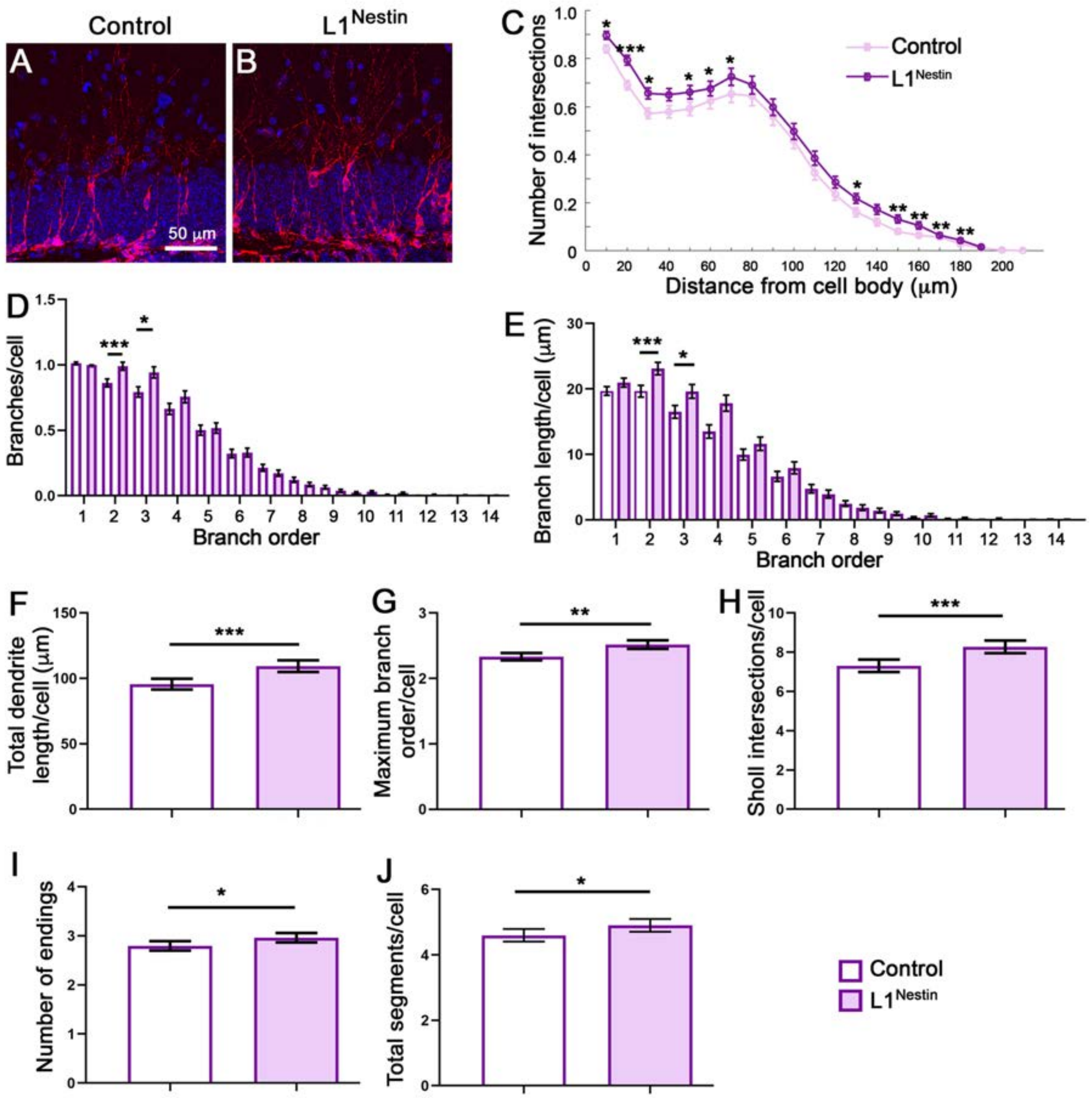


**Figure S6. Deletion of *L1cam* results in dendrites with increased branching in L1$^{Nestin}$ mutants. Related to Figure 5.** Sections of control **(A)** and mutants **(B)** were immunostained for DCX (red) and DAPI (blue). **(C)** Sholl analysis was performed showing the number of intersections over the distance from the cell body. *p* values for significant differences are: 10 µm, **p* = 0.0224; 20 µm, ****p* = 0.0010; 30 µm, **p* = 0.0171; 50 µm, **p* = 0.0291; 60 µm, **p* = 0.0393; 70 µm, **p* = 0.0363; 130 µm, **p* = 0.0371; 150 µm, ***p* = 0.0011; 160 µm, ***p* = 0.0037; 170 µm, ***p* = 0.0027; 180 µm, ***p* = 0.0086. N = 4 mice and n = 1,344 cells for controls; N = 4 mice and 1,056 cells for mutants. **(D)** Number of branches by branch order per cell. ****p* = 0.0007; **p* = 0.0419. **(E)** Branch length by branch order per cell. ****p* = 0.0006 ($2^o$ branch); **p* = 0.0435 ($3^o$ branch). The number of mice and cells analyzed in (D) and (E) are as in (C). For (D) and (E), a one-way ANOVA was performed, mean ± SEM. **(F)** Total dendrite length per cell. ****p* = 0.0007. N = 4 mice and 1,069 cells for controls and 4 mice and 1,057 cells for

mutants. **(G)** Maximum branch order per cell. ** $p$ = 0.0033. Number of mice and cells are as in (C). **(H)** Sum of Sholl intersections per cell. *** $p$ = 0.0004. N = 4 mice and 1066 cells for controls and 4 mice and 1,152 cells for mutants. **(I)** Number of endings. * $p$ = 0.0117. N = 4. **(J)** Total segments per cell. * $p$ = 0.0153. N = 4. **(F-J)** Mann-Whitney test, mean ± SEM.

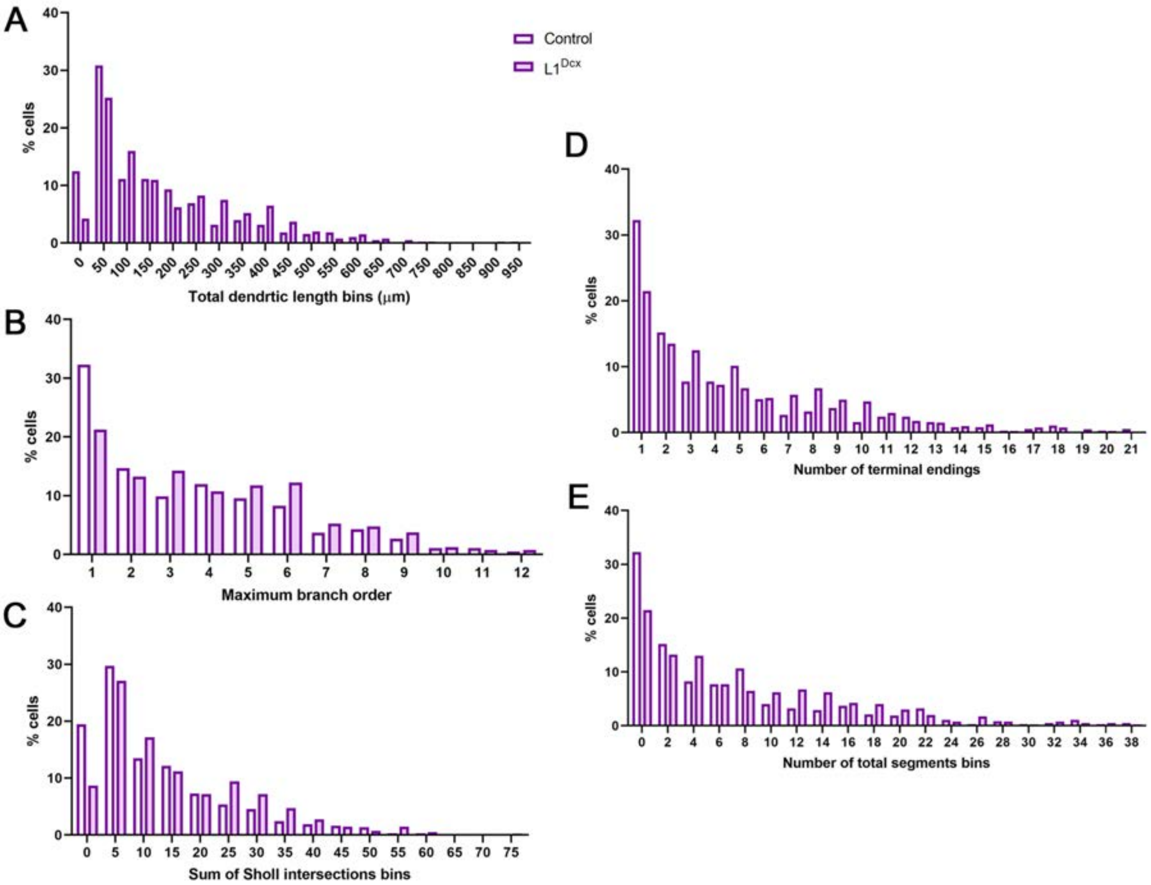


**Figure S7. Cell distributions for $L1^{Dcx}$ dendrite analyses. Related to Figure 5. (A)** Distribution of cells grouped in 50 µm bins according to their total dendritic length. (**B)** Distribution of cells grouped according to the maximum branch order they reached. **(C)** Distribution of cells grouped in bins according to the sum of their total Sholl intersections. **(D)** Distribution of cells by total number of terminal endings. **(E)** Distribution of cells grouped according to their total number of segments. See Figure 5 for numbers of mice and cells used in each analysis.

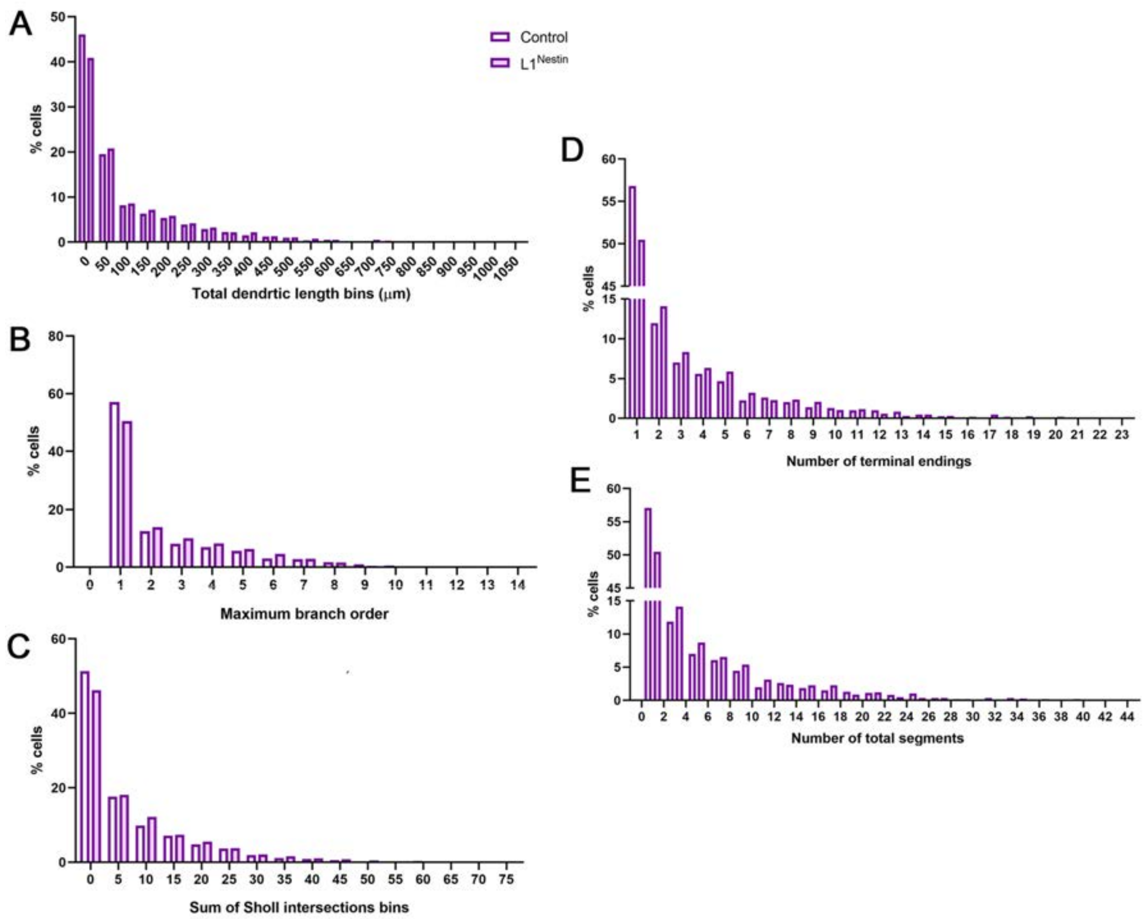


**Figure S8. Cell distributions for L1$^{Nestin}$ dendrite analyses. Related to Figure 5 and S6. (A)** Distribution of cells grouped in 50 μm bins according to their total dendritic length. (**B)** Distribution of cells grouped according to the maximum branch order they reached. **(C)** Distribution of cells grouped in bins according to the sum of their total Sholl intersections. **(D)** Distribution of cells by total number of terminal endings. **(E)** Distribution of cells grouped according to their total number of segments. N = 4 mice and 1,070 cells for control and 4 mice and 1,056 cells for mutants.
